# 1Differential immune mechanism to HIV-1 Tat variants and its regulation by AEA

**DOI:** 10.1038/srep09887

**Published:** 2015-05-06

**Authors:** Gopinath Krishnan, Nivedita Chatterjee

**Affiliations:** 1 L&T Department of Ocular Pathology, Vision Research Foundation, Sankara Nethralaya, 41 College Road, Chennai, 600006 India; 2 Research Scholar, CeNTAB, School of Chemical and Biotechnology, SASTRA University, Tanjore, India

## Abstract

In the retina, Müller glia is a dominant player of immune response. The HIV-1 transactivator viral protein (Tat) induces production of several neurotoxic cytokines in retinal cells. We show that HIV-1 clades Tat B and C act differentially on Müller glia, which is reflected in apoptosis, activation of cell death pathway components and pro-inflammatory cytokines. The harsher immune-mediated pathology of Tat B, as opposed to milder effects of Tat C, manifests at several signal transduction pathways, notably, MAPK, STAT, SOCS, the NFκB signalosome, and TTP. In activated cells, anandamide (AEA), acting as an immune-modulator, suppresses Tat B effect through MKP-1 but Tat C action via MEK-1. AEA lowers nuclear NF-κB and TAB2 for both variants while elevating IRAK1BP1 in activated Müller glia. Müller glia exposed to Tat shows enhanced PBMC attachment. Tat-induced increase in leukocyte adhesion to Müller cells can be mitigated by AEA, involving both CB receptors. This study identifies multiple signalling components that drive immune-mediated pathology and contribute to disease severity in HIV clades. We show that the protective effects of AEA occur at various stages in cytokine generation and are clade-dependant.

Several reports have implicated specific Human immunodeficiency virus-1 (HIV1) subtypes (clades) with an accelerated disease progression compared to other HIV1 clades^1^ in socio-economically and geographically- matched patients. This is connected to widespread neuro-inflammation in the central nervous system caused by the whole virus or viral proteins shed from infected cells. The role of two HIV proteins, HIV-1 Tat (transactivator viral protein) and gp120, in neuro-inflammation have been extensively studied. Cells produce Tat, a key component of the virus coat, early in infection. It is pleiotropic in character and is involved in different aspects of AIDS pathogenesis. Tat not only plays an important role in viral transcription and replication, but can also induce the expression of a variety of cellular host genes^2^. It has been shown to cause cell death by affecting caspase levels, dissipating mitochondrial membrane potential^3^ and causing cytochrome c release from tissues. Out of the 11 clades (A-K), Tat protein from clade B shows more neurotoxic effect and consequent clade-specific differences in neuropathogenesis and cognitive dysfunction^4^. Additionally, Tat from clade B is known to be highly effective in inducing inflammatory chemokines in astrocytes^5^,^6^,^7^ and microglia^8^. An excitotoxic and inflammatory response from glia has been linked to neurodegeneration^9^. Lesser neurotoxic properties and chemotactic ability of HIV-1 Tat C has been attributed to differences in protein structure and sequence from Tat B^3^. However this structural difference does not affect its transactivation properties^10^,^11^,^12^. Reports on how Tat B and Tat C act on the signal transduction machinery involved in the inflammatory process remain lacking in the literature to date.

Individuals with HIV-1 often suffer from visual impairment and opportunistic retinitis^13^. While anti-retroviral therapies have reduced the incidence of infections such as CMV, complications to the visual system persist. The predominant glial cells in the retina, the Müller glia, are actively involved in many inflammatory conditions. Activation of Müller glia has an innate immune component^14^,^15^. Importantly, Müller glia takes up many of the functions of astrocytes and microglia in the brain^16^ and make up part of the inner blood retinal barrier (BRB). We look at Müller glia of the retina in the context of HIV-1 Tat induced inflammation. While we have used human retinal primary Müller glia in this study as a model system, the results are likely to be valid in any glial cells activated by HIV-1. We investigate differences in innate immune response and ability to attract monocytes on exposure to clade B and clade C HIV-1 Tat in retinal Müller glia.

The endocannabinoid system is known to aid neuronal survival in several neurodegenerative disorders^17^. Endocannabinoids are being increasingly explored as immune-modulators. They suppress inflammation by inhibiting release of inflammatory mediators. Previous reports suggest that cannabinoids are able to suppress pro-inflammatory factors and macrophage migration induced by HIV Tat protein^18^,^19^,^20^. Endocannabinoid therapy may be particularly valuable where secondary inflammatory damage can cause a neurodegenerative cascade^21^ and compromise the blood brain barrier (BBB). HIV-1 invades the central nervous system through the transmigration of infected monocytes across the BBB^22^. Several studies have investigated leukocyte-endothelial cell interaction in the context of cannabinoid therapy^23^. Little is known about how engagement of the cannabinoid receptors affect the activated state of the Müller glia which participate in blood-retinal barrier functions. We report that (1) Tat variants act to produce cytokines through different components of the MAPK, STAT and SOCS pathway, (2) N-arachidonoylethanolamide (AEA) treatment, which promote anti-inflammatory milieu in cells exposed to either Tat variants, mediate this response through differential action on these pathways, and (3) monocyte adhesion can be diminished on exposure to AEA for both clades but through changes in expression of MEK-1 for Tat C and Mitogen-activated protein kinase phosphatase-1 (MKP-1) for Tat B. Our work shows that in the retina, regulation of Müller glial innate immune response, ability to attach leukocytes and the machinery involved in cytokine production is clade specific.

## Results

### AEA rescues cell death induced by Tat B and Tat C and suppresses pro-inflammatory cytokines

**Figure 1:**
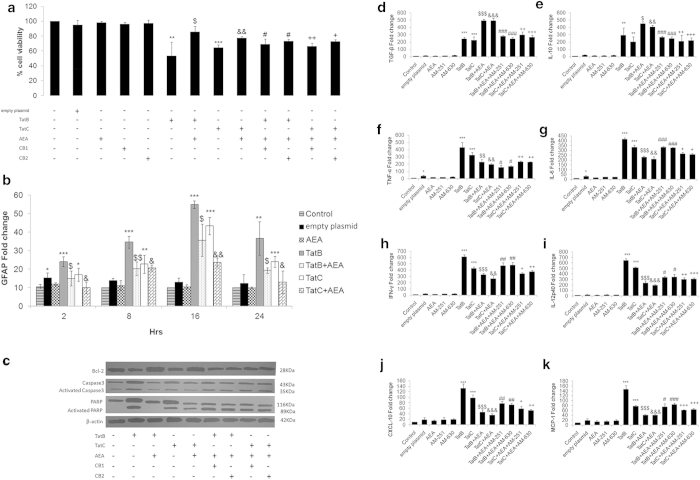
**AEA affects cell death pathway components, GFAP activation and cytokine production, induced by either Tat B or C.** Both Tat B and Tat C reduce cell viability in primary Müller glia. Addition of AEA to Tat-activated cells increases cell viability. CB1 and CB2 antagonists partially reverse the action of AEA (**a**). GFAP mRNA fold increase on expression of Tat B/C plasmids. Tat alone induced GFAP at all time points with peak at 16 hours. Addition of AEA in Tat B/C transfected cells reduced GFAP levels (**b**). Bcl-2 levels increase in activated cells on exposure to AEA. Cropped immunoblots for apoptotic and necrotic markers, Caspase-3 and PARP respectively, were seen to increase on Tat B/C transfection. AEA treatment with Tat B/C lowered Caspase-3 and PARP levels. CB1/2 antagonists with Tat B/C + AEA showed reversal in Caspase-3 and PARP expression (**c**). Full-length blots are presented in Supplementary Fig. S6. Histograms are given as mean ± S.E. (n = 3); *p < 0.05, **p < 0.01, ***p < 0.001 vs. Control. $p < 0.01, $$p < 0.01 vs Tat B. &p < 0.05, &&p < 0.01 vs Tat C. #p < 0.05 vs. Tat B + AEA. + p < 0.05, ++ p < 0.01 vs. Tat C + AEA. Real Time PCR quantitative analysis showed significant increase in TGF-ß and IL-10 (**d**, **e**) when Tat transfected cells were incubated with AEA. Fold change of TNF-α, IL-6, IFN-γ, IL-12, CXCL- 10, and MCP-1 (**f**-**k**) showed suppression of their mRNA in activated Müller glia exposed to AEA. CB1/CB2 inhibitors reversed the effects of AEA partially. AEA effect on suppressing pro- inflammatory cytokines is more noticeable in Tat B than in Tat C cells. Cells were transfected with Tat B or Tat C alone or transfected cells exposed to AEA for 8-10 hours. Cells transfected with empty plasmid or AEA and the CB antagonists alone showed nominal or no change in cell viability (**a**), GFAP expression (**b**), and the aforementioned cytokines (**d**-**k**).Values are given as mean ± S.E (n = 3). *p < 0.05, **p < 0.01, ***p < 0.001 vs. Control. $p < 0.001, $$p < 0.01,$$$p < 0.001vs Tat B. &&p < 0.01, &&&p < 0.001 vs Tat C. #p < 0.05, ## < 0.01, ###p < 0.001 vs Tat B + AEA. +p < 0.05, ++p< 0.01, +++< 0.001 vs. Tat C + AEA.

It has been reported that differential neuropathological conditions exist in neurons on exposure to Tat B and Tat C clades^4^. In primary human Müller glia we find that transfection of Tat B or Tat C reduces cell viability to 54 and 61%, respectively. Addition of AEA to Müller glia cultures in cells transfected with Tat B or Tat C significantly increases cell viability to 85 and 79%, respectively. Cannabinoid receptor (CB) blockers are seen to reverse the effect of AEA treatment significantly (Fig. 1a). We find that Tat B/C transfection significantly increases levels of GFAP, an activation marker in Müller glia^24^, with peak at 16 hours (Fig. 1b). Addition of AEA to Tat B or Tat C cells reduces GFAP level. Bcl-2 is a survival factor and AEA treatment increases its expression. In contrast, Tat B/C transfected cells show very low Bcl-2 expression. To evaluate the cell death pathway, we assayed for cleaved caspase-3 and PARP levels. Cleaved caspase-3 and PARP are highly expressed on Tat B or Tat C transfection. Their expressions are lower on AEA treatment. Addition of CB1/2 inhibitors reverses the effect of AEA (Fig. 1c).

HIV-1 Tat is known to induce neurotoxic cytokines^25^. We quantified several cytokines by qPCR (Fig. 1 d-k). While the level of pro-inflammatory cytokines, TNF-α, IL-6, IFN-γ and IL-12 (Fig. 1 f-k) are characteristically higher in Tat B cells compared to Tat C cells, anti-inflammatory IL-10 and TGF-β (Fig. 1 d, e) show less variation. AEA can regulate the level of cytokine production in Müller glia activated by both Tat B and Tat C. CXCL-10 and MCP-1 measured at 10 hours increase in the presence of Tat B and to a lesser extent in Tat C cells, and show suppression on exposure to AEA (Fig. 1 j, k). Initial experiments established concentration of AEA used (10 μM) and time points for assaying cytokine mRNA and protein (Supplementary Fig. 1C-F). After 8 hours of transfection, both Tat B and Tat C produce peak levels of all cytokines tested compared to control. CB1/2 blockers reverse effects of AEA on cytokine mRNA production (Fig. 1 d-k). Cell viability did not change at all with backbone plasmid or any of the treatment reagents alone (a). Cytokine production and GFAP levels also remained largely unchanged (b-k).

### MAPK phosphorylation is reduced in Tat B and Tat C transfected cells exposed to AEA

**Figure 2:**
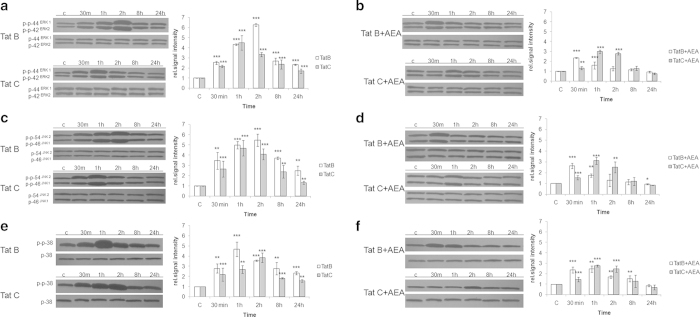
**AEA suppresses MAPK phosphorylation in either Tat B or Tat C transfected Müller glia.** Tat B transfected (**a**) Müller glia shows peak phosphorylation of ERK1/2 and JNK, at 2 hours, but at 1 hour in Tat C (**b**) cells. Peak phosphorylation of p38 occurred at 1 hour in Tat B cells but at 2 hours in Tat C cells (**a** and **b**). In AEA exposed cells, peak phosphorylation occurs earlier on transfection with Tat B than in Tat C. Levels of phosphorylation were suppressed in AEA treated cells relative to Tat alone, for both variants. Normalized levels of phosphorylated ERK1/2, JNK, p38 are shown as mean ± S.E (n = 3). Representative blots shown. Representative blots were cropped. Full-length blots are presented in Supplementary Fig. S6. *p < 0.05, **p < 0.01, ***p < 0.001 vs. Control.

MAPK activation is a key pathway in inflammatory conditions. Transfection with Tat B induces peak phosphorylation in ERK1/2 and JNK at 2 hours (Fig. 2a, c). Tat B and Tat C both act on MAPK components, but show differences in magnitude and temporal pattern. Tat C transfection alone induces peak phosphorylation of ERK, and JNK at 1 hour. While peak p38 levels occur at 1 hour in Tat B cells, in Tat C cells it is at 2 hours (Fig. 2 a, c, e). Co-incubation with AEA in transfected cells magnifies the differential effect on MAPK. AEA exposure reduces both duration and level of phosphorylation. An early phosphorylation (30 minutes) is followed by gradual decrease in Tat B cells exposed to AEA (Fig. 2 b, d, and f). This fall does not occur in Tat C cells + AEA. Thus, at 1 and 2 hours, phosphorylation levels of ERK and JNK in Tat C cells are less affected on exposure to AEA. From 8 hours onward phosphorylation levels are similar in both Tat B/C cells. It is important to note that peak phosphorylation in AEA treated cells occur earlier in Tat B cells compared to Tat C glia. Empty plasmid or AEA alone did not induce ERK1/2 phosphorylation (see Supplementary Fig. S2 online).

We also dissected the pI3/AKT pathway implicated in cytokine generation^26^,^27^. Tat B transfection induces AKT phosphorylation at 24 hours to a lesser extent than on Tat C transfection. Addition of AEA with Tat B/C reduces phosphorylation levels of AKT at 24 hours. Treatment with CB1/2 blockers partially reverse these effects (see Supplementary Fig. S3 online).

### AEA affects Suppressor of Cytokine Signalling (SOCS) and STAT-1α in Tat B/C cells in the late phase of activation

**Figure 3:**
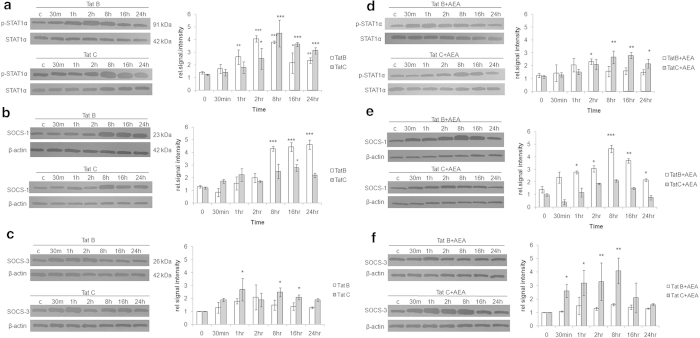
**The STAT-SOCS feedback loop is regulated in a clade specific manner by AEA in activated Müller glia.** Tat B and Tat C transfection affect STAT and SOCS in a differential manner. STAT levels are low when SOCS is high for both Tat variants, 8 hours onward (**a**, **b**). SOC-3 levels show negligible variation in either Tat variant, alone (**c**). AEA treatment lead to suppression of STAT levels for both variants (**d**). Exposure to AEA in Tat B cells lead to rise in SOCS-1 level, 30 min onward (**e**). Tat C + AEA treatment showed negligible effect on levels of SOCS-1 but increase in SOCS-3, 30 min onward (**f**). Normalized levels of phosphorylated STAT-1α, SOCS-1 and SOCS-3 are shown as mean ± S.E (n = 3). Representative blots shown. Representative blots were cropped. Full-length blots are presented in Supplementary Fig. S6. *p < 0.05, **p < 0.01,***p < 0.001 time points vs. 0 min.

In monocytes it has been previously demonstrated that Tat B impaired the IFN gamma-receptor mediated transcription of specific genes at the level of STAT1 activation^28^. STAT and SOCS levels contribute to pro-inflammatory cytokine levels through negative feedback^29^. STAT, stimulated on production of cytokines, induce SOCS. This in turn negatively regulates cytokine signalling. STAT1α is conspicuously higher at the later phase in Tat C cells, while in Tat B cells it falls after 2 hours. With SOCS1, this classical negative feedback loop is very obvious on transfection with either Tat B or Tat C (Fig. 3 a, b). SOCS3 levels change only marginally with either Tat plasmid (Fig. 3c). Thus, Tat B/C influences STAT and SOCS in a differential manner. On addition of AEA, phosphorylation of STAT is suppressed in Tat B/C cells. Again this is more noticeable in the later phase of activation. SOCS1 rises dramatically in Tat B + AEA cells. Normally, for this feedback pathway to be operational, an increase in cytokines results in a gradual rise in STAT-1α. However, here we observe that AEA induces SOCS in Tat B cells despite the overall lesser amount of cytokines (Fig. 1 d-k) and STAT1α (Fig. 3 d, e). This is likely to dampen production of pro-inflammatory cytokines. We find SOCS3 is affected by Tat C, but not Tat B, in the presence of AEA (Fig. 3f). Empty plasmid or AEA alone did not change the phosphorylation levels of STAT-1α, SOCS-1, and SOCS-3 (see Supplementary Fig. 2 online).

### Pro-inflammatory cytokine production and regulation by AEA is dependent on MAPK phosphorylation in Tat-induced Müller glia

**Figure 4:**
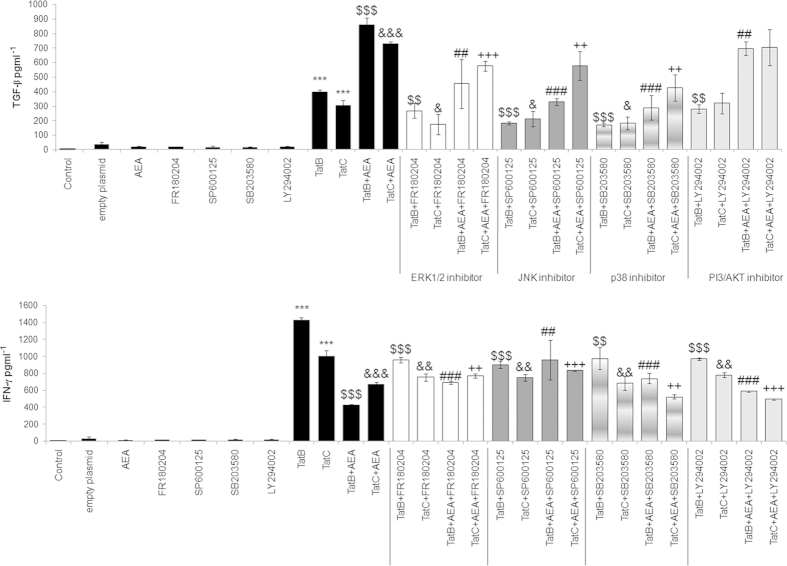
**AEA affect cytokine generation on blockage of MAPK components.** Transfection of Tat B or Tat C increased TGF-β production significantly compared to control cells. Addition of AEA to Tat B/C transfected cells significantly increased the production of TGF-β compared to Tat B/C transfection alone. Tat B/C + AEA cells were treated with pharmacological inhibitors for ERK1/2, JNK, p38 signalling; FR180204, SP600125, and SB203580, respectively. Addition of ERK, JNK, p38 inhibitors significantly down regulated the production of TGF-β compared to Tat B/C + AEA treated cells (**a**). IFN-γ was upregulated on transfection with Tat B/C compared to control cells. Addition of AEA to Tat B/C cells significantly reduced IFN-γ production compared to Tat B/C cells. Incubation of Tat B/C + AEA cells with MAPK blockers significantly increased IFN-γ, the exception being on addition of SB203580 (**b**). PI3/AKT inhibition affects production of IFN-γ but not of TGF-β in Tat C + AEA cells. Transfection of empty plasmid or AEA, inhibitors to the MAPK enzymes and pI3-AKT blocker alone do not increase production of TGF-β or IFN-γ (**a**, **b**). Results shown are the mean ± S.E (n = 3). ***p < 0.001 vs. Control. $$p < 0.01, $$$p < 0.001 vs Tat B. &p < 0.05, &&p < 0.01, &&&p < 0.001 vs Tat C. ##p < 0.01, ###p < 0.001 vs. Tat B + AEA. ++ p < 0.01, +++< 0.001 vs. Tat C + AEA.

Contribution to cytokine production of the several pathways dissected in this study has been further tested by using inhibitors. Addition of inhibitors, FR180204, SP600125, SB203580 against ERK1/2, JNK, p38 respectively, with either Tat variants and AEA , increases production of pro-inflammatory cytokines, IFN-γ (Fig. 4b), TNF-α, IL-6, IL-12p70, CXCL-10, and IL-8 (see Supplementary Fig. S4 online). Anti-inflammatory cytokines TGF-β (Fig. 4a) and IL-10 (see Supplementary Fig. S4 online) levels reduce on inhibiting these pathways with either variant of Tat and AEA. Inhibition of PI3/AKT showed negligible effect except for IFN-γ (Fig. 4b), IL-6 and CXCL-10 (see Supplementary Fig. S4 online).Therefore, cytokine production induced by both Tat variants is primarily through MAPK. Transfection with the empty plasmid, AEA, inhibitors to MAPK enzymes and pI3-AKT blocker alone do not increase production of the cytokines (Fig. 4a, b, Supplementary Fig. S4 online).

### Tat C induced inflammation is regulated by AEA through MEK

**Figure 5:**
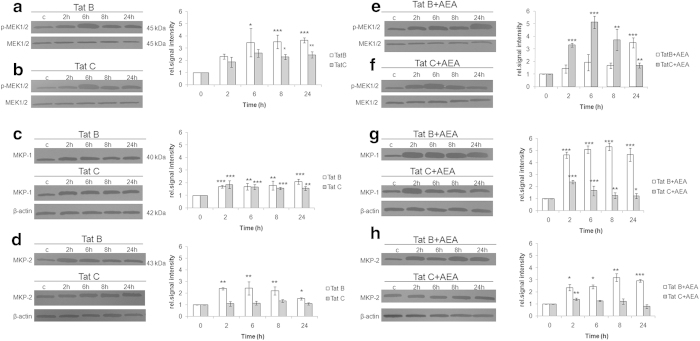
**AEA acts in Tat B transfected cells through MKP-1 but in Tat C transfected cells through MEK.** Tat B transfection induced sustained and elevated phosphorylation of MEK from 6 hours onward (**a**). Tat C induced phosphorylation of MEK was lower at all time points and fell after reaching peak at 6 hours (**b**). Effect of AEA in Tat C cells on the phosphorylation of MEK was more pronounced than on Tat B cells (**c**). Both Tat variants induced MKP-1 level 2 hours onward (**e**). Increase in MKP-2 occurs only with Tat B but not Tat C (**f**). AEA exposure affects MKP-1levels in a sustained manner in Tat B cells, while in Tat C + AEA cells, MKP levels rise early and fall thereafter (**g**). AEA has pronounced effect on Tat B cells but not Tat C cells (**h**). Normalized levels of phosphorylated MEK-1/2 with MEK-1/2 and MKP-1 as well as MKP-2 with β-actin are shown as mean ± S.E (n = 3). Representative blots shown. Representative blots were cropped. Full-length blots are presented in Supplementary Fig. S6. *p < 0.05, **p < 0.01, ***p < 0.001 vs 0 min.

Phosphorylation of MAP kinase components are controlled by a battery of kinases and phosphatases^30^,^31^. *Mitogen-activated protein kinase* kinase (MEK), a MAP2K regulator show phosphorylation 2 hours onward on transfection with Tat B (Fig. 5a). Transfection with Tat C also induced the phosphorylation of MEK from 2 hours onward but to a lesser extent (Fig. 5b). Even though MEK-1/2 remains high in activated cells at later time points, phosphorylation of MAPK continues to fall (Fig. 2). We have, therefore, analysed the role of the phosphatase, MKP-1/2. MKP-1 gradually increases on transfection of Tat B/C. Noticeably, effect of Tat C on MKP-2 is nominal (Fig. 5c, d). Addition of AEA in Tat C-transfected cells shows a striking increase in MEK levels up to 8 hours (Fig. 5f). Cells with Tat B + AEA show significant rise in MEK levels only at 24 hours (Fig. 5e). In contrast, MKP-1/2 are highly induced on stimulation with Tat B + AEA and consistently maintained (Fig. 5g, h). Tat C + AEA treatment shows a brief rise in MKP-1/2 (Fig. 5g, h). Thus, in the presence of AEA, Tat B action is mediated through MKP-1/2 but Tat C action is mediated by MEK-1. This is further confirmed by inhibitor studies. Transfection with empty plasmid or AEA alone did not induce phosphorylation of MEK1/2, MKP-1 and MKP-2 (see Supplementary Fig. 2 online).

### MEK is required for AEA regulation of Tat C induced MAPK phosphorylation

**Figure 6:**
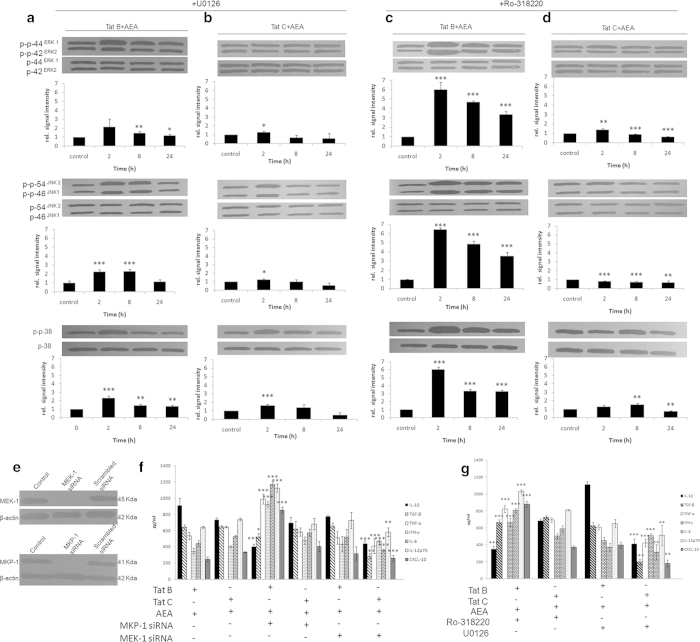
**AEA action on MAPK activation is mediated through MEK-1 in Tat C cells, but not in Tat B cells.** MEK inhibition by U0126 increases MAPK phosphorylation in Tat C + AEA, but to a lesser extent in Tat B + AEA. In both Tat B and Tat C cells, AEA induces peak phosphorylation at 2 hours (**a**, **b**). On exposure to AEA, MKP inhibition by Ro-318220 increase MAPK phosphorylation significantly more in Tat B cells (**c**) than in Tat C cells (**d**). Representative blots shown. Normalized levels of phosphorylated ERK1/2, JNK, p38 are shown as mean ± S.E (n = 3). *p < 0.05, **p < 0.01,***p < 0.001 vs. Control. Cropped Western blot data show silencing with MEK-1 or MKP-1 siRNA block MEK-1 or MKP-1 expression (**e**). Full-length blots are presented in Supplementary Fig. S6. On MKP-1 siRNA treatment of Tat B + AEA cells, there is suppression in TGF-β and IL-10. TNF-α, IFN-γ, IL-6, IL-12p70 and CXCL-10 are however elevated. Tat B + AEA treated cells with MEK-1 knockdown showed comparable levels of cytokines relative to Tat B + AEA only (**f**). MEK-1 siRNA in Tat C + AEA cells significantly reduced the levels of pro- inflammatory cytokines compared to Tat C + AEA only cells. Tat C + AEA cells with MKP-1siRNA showed similar profile to Tat C + AEA only cells (**f**). Treatment by pharmacological blockers for MKP-1 and MEK-1 in Tat B + AEA or Tat C + AEA cells show results similar to siRNA knockdown (**g**). ELISA was used to measure cytokine protein levels. Results shown are the mean ± S.E (n = 3). *p < 0.05, **p < 0.01, ***p < 0.001 vs. Tat B + AEA.

The rival roles of MEK-1 and MKP-1 in the AEA-mediated regulation of Tat B/C activated Müller glia has been explored further. To dissect AEA induced phosphorylation of MAPK, we use MEK blocker U0126 in Tat B + AEA or Tat C + AEA cells. We find that blocking MEK in Tat C + AEA cells reduces phosphorylation levels of MAPK (Fig. 6b) much more than in Tat B + AEA cells (Fig. 6a). Blocking MKP-1 increases phosphorylation levels only in Tat B + AEA (Fig. 6c) cells. MKP-1 blockage has pronounced effect in Tat B + AEA cells and less in Tat C + AEA (Fig. 6d). These observations emphasise that exposure to AEA in presence of Tat B or Tat C regulates phosphorylation of MAPK differentially, thus engendering cytokine production at very different levels.

### AEA-mediated induction in MEK is sufficient in suppressing pro-inflammatory and increasing anti-inflammatory cytokines in Tat C transfected Müller glia, but not in Tat B cells

To test the role of MKP-1 and MEK-1 in the cytokine production pathway, cells were either first treated with MKP-1, MEK-1 siRNA or scrambled siRNA. Western blot data show MEK-1 or MKP-1 siRNA blocks MEK-1 or MKP-1 expression (Fig. 6e) On MKP-1 knockdown, Tat C + AEA cells show negligible differences from control cells exposed to scrambled siRNA in the profile of all the cytokines studied. MKP-1 silencing in Tat B + AEA cells however show pronounced suppression in production of anti-inflammatory cytokines and increase in pro-inflammatory ones. MEK-1 siRNA transfection in cells with Tat B + AEA shows similar production of cytokine levels compared to control cells. Silencing MEK-1 in cells transfected with Tat C and exposed to AEA results in decreased production of all cytokines investigated in this study, suggesting a critical role of MEK-1 (Fig. 6f). Inhibition of MKP-1 and MEK-1 by pharmacological blockers show similar results (Fig. 6g). The role of MEK-1 in generation of cytokines induced by Tat C is also reiterated when we block MEK-1 in the presence of Tat C alone. The relative importance of MKP-1 and MEK-1 is also validated after inhibiting these enzymes and ascertaining mRNA level of the cytokines IL-10, and MCP-1, at 8 and 24 hours (see Supplementary Fig. S5 online) MKP-1, MEK-1 scrambled siRNA did not show any difference from Tat C + AEA, Tat B + AEA cells respectively.

### Tat B and Tat C induced inflammation is regulated in a differential manner by AEA at the NF-κB signalosome

**Figure 7:**
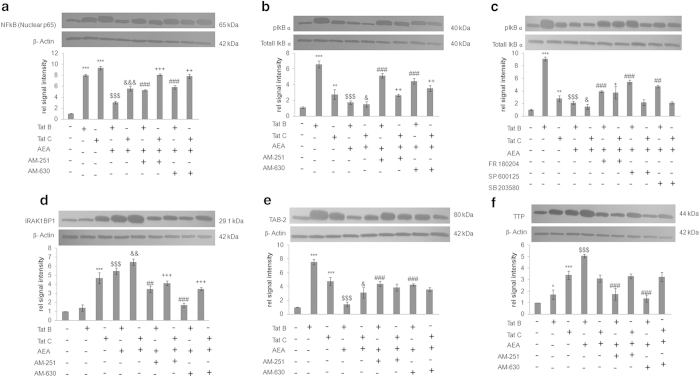
**Tat B and Tat C differentially affect NF-kB signalling.** Nuclear NF-κB significantly increased on transfection with Tat B or Tat C. Addition of AEA significantly reduced the expression of nuclear NF-κB levels. Addition of CB1/2 blockers reversed the effect of AEA on Tat B/C transfected cells (**a**). Phosphorylated IκB increased enormously on transfection of Tat B. On transfection with Tat C, pIkB expression showed mild increase. AEA on Tat B cells reduced the expression of pIκB. Nominal down regulation of pIκB was seen in Tat C cells with AEA. Incubation with CB blockers partially reverses AEA effect (**b**). Inhibition with MAPK blockers significantly reduced Tat B + AEA effect on pIκB. In Tat C + AEA cells, significant reversal of AEA effects on pIκB was observed only on blocking with ERK inhibitor FR180204 (**c**). IRAK1BP1 levels increased significantly on Tat C transfection but not Tat B. Incubation with AEA on Tat B/C cells significantly increased IRAK1BP1 levels. Reversal by CB blockers of AEA effects was more pronounced for Tat B + AEA (**d**). TAB-2 increased with Tat B/C transfection. Addition of AEA reduces TAB2 significantly. Addition of CB blockers partially reverses effect on Tat B cells but not Tat C cells (**e**). TTP level increased significantly on transfection of Tat C. Addition of AEA increased TTP significantly only in Tat B cells. (**f**). Addition of CB blockers reverse AEA effect on Tat B cells but not in Tat C cells. Representative blots shown. Representative blots were cropped. Full-length blots are presented in Supplementary Fig. S6. Results shown are the mean ± S.E (n = 3). *p < 0.05, **p < 0.01, ***p < 0.001 vs. Control. $$$p < 0.001 vs Tat B. &p < 0.05, &&p < 0.01, &&&p < 0.001 vs Tat C. ##p < 0.01, ###p < 0.001 vs. Tat B + AEA. + p < 0.5, ++ p < 0.01, +++ < 0.001 vs. Tat C+AEA.

AEA has already been implicated in cytokine production in microglia and Müller glia by regulating phosphorylation of NF-κB^32^,^12^. We look at several components comprising the NF-κB transcription machinery. Both Tat B and Tat C increase p65 levels in the nucleus. AEA exposure reverses this effect. That AEA action is indeed mediated by the CB receptors is shown by the increased levels of p65 NFκB in the presence of (N-(Piperidin-1-yl)-5-(4-iodophenyl)-1-(2,4-dichlorophenyl)-4-methyl-1H-pyrazole-3 carboxamide) AM-251 and (6-Iodo-2-methyl-1-[2-(4-morpholinyl)ethyl]-1H-indol-3-yl](4-methoxyphenyl)methanone) AM-630 (Fig. 7a). Nuclear NF-κB levels are proportional to phosphorylated IκBα in the cytoplasm. Thus, when AEA exposure leads to high levels of pIκBα (Fig. 7b) in Tat B cells, NF-κB will remain in the cytoplasm. In Tat C cells, increase in cytoplasmic pIκBα is nominal. AEA exposure leads to similar levels of pIκBα regardless of the clade. The intimate link of the MAPK pathway with the NF-κB signalosome is further proved by using inhibitors against MAP kinases and investigating how pIκBα changes. Only on inhibition of ERK do we observe an appreciable increase in the reversal of pIκBα levels in Tat C + AEA cells. By contrast, inhibitors to all the MAP kinases affect Tat B + AEA induced suppression of pIκBα (Fig. 7c). The NF-κB complex is composed of multiple molecules which aid in orchestrating a balanced innate immune response, sufficient for defence while avoiding excessive detriment to host tissues. IL-1R-associated kinase 1 binding protein 1 (IRAK1BP1) has been identified as playing an inhibitory response during inflammation^33^. Tat C transfected cells show a remarkable increase of IRAK1BP1, much more than Tat B cells. In the presence of AEA, the increase in IRAK1BP1 levels seen in Tat B cells is therefore more significant than in Tat C cells. AEA action is mediated by the CB receptors as shown by the decreased levels in the presence of AM-251 and AM-630 (Fig. 7d). Inflammatory responses have also implicated TAK1 and its associated adaptor protein TAB2^34^,3^35^. AEA action through MAPK and NF-κB in activated Müller glia prompted us to explore TAB2 levels. TAB2 levels increase in the presence of both Tat variants. In Tat C cells, the expression is milder than in Tat B cells. AEA suppresses TAB2 levels for both Tat B/C. However, the reversal of AEA effect is more noticeable in Tat B cells than in Tat C cells, as judged by inhibitors to CB1/2 (Fig. 7e). Further dissection of signalling pathways affecting cytokine levels show that, in addition to controlling NF-κB, AEA may affect innate immune response by altering cytokine mRNA stability. Presence of AU-rich elements (ARE) at the 3’ untranslated regions is seen in pro-inflammatory cytokines such as IL-6. Tristetraprolin (TTP) can bind to and influence stability of ARE containing transcripts^36^. Tat C induces higher expression of TTP than Tat B. Therefore, exposure to Tat C would likely destabilise more pro-inflammatory cytokine mRNA. AEA causes significant rise in TTP levels in Tat B cells (Fig. 7f). In Tat C cells, AEA has negligible effect. AEA action is more prominent in increasing TTP levels in cells transfected with Tat B as ascertained by CB receptor inhibition. Transfection with empty plasmid or exposure to AEA, and the CB blockers alone do not change the expression of nuclear NF-κB, pIκB, IRAK1BP1, TAB2, and TTP. Similarly, pIκB expression does not increase on treatment with MAPK blockers alone compared to untreated cells (see Supplementary Fig. 2). We further examine how AEA can affect the state of Müller activation in the context of PBMC attachment.

### PBMC adhesion to Müller glia can be regulated at the level of MKP-1 and is clade-dependent

**Figure 8:**
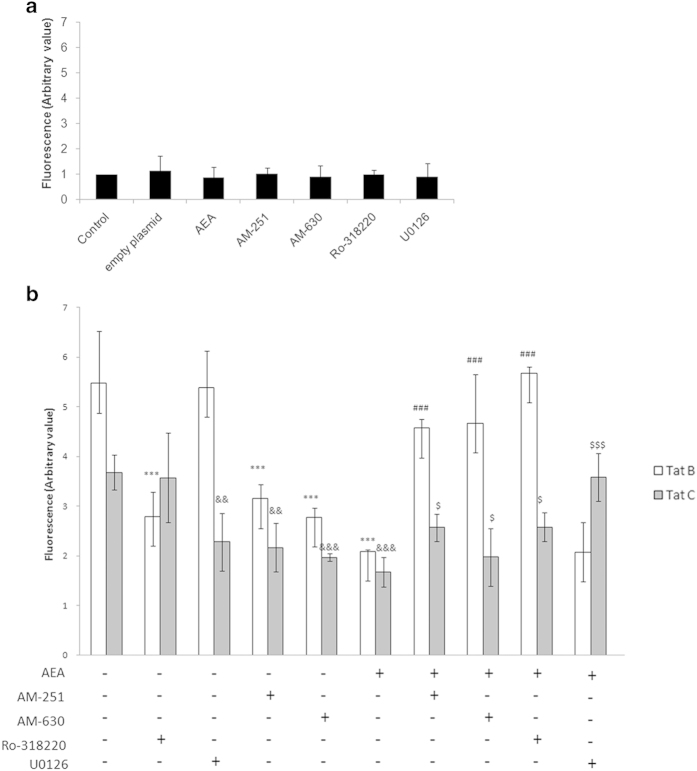
**Tat-induced adhesion of PBMC to Müller cells is inhibited by AEA.** Tat B or Tat C induced PBMC adhesion to Müller cell line MIO-M1. Antagonists U0126, Ro- 318220, AM-251 and AM-630 alleviated PBMC adhesion, but not to the extent of AEA. CB blockers reversed the effect of AEA and increased PBMC adhesion. MKP-1 inhibition has a striking effect on Tat B + AEA cells but nominally affects Tat C + AEA cells compared to either Tat B + AEA or Tat C + AEA. MEK-1 inhibition affects adhesion to a significant extent only in Tat C + AEA cells, but not Tat B + AEA. MIO-M1 cells were transfected with Tat B or Tat C and then treated with AEA. MEK-1 or MKP-1 blocker (U0126 and Ro-318220, respectively), CB blockers (AM-251 or AM-630) were used simultaneously for 24 hours, wherever used (**b**). Adherent cells were expressed as arbitrary values. Transfection with empty plasmid or AEA and blockers to CB receptors, MEK-1, and MKP-1 alone do not induce PBMC adhesion to MIO-M1 (**a**). Results are expressed as mean ± S.E (n = 3). ***p < 0.001 vs Tat B, &&p < 0.01, &&&p < 0.001 vs Tat C, ###p < 0.001 vs Tat B + AEA, $p < 0.05, $$$p < 0.001 vs Tat C + AEA.

Engagement with CB receptors, particularly CB2 is known to inhibit macrophage adhesion^37^ and subsequent migration^23^. Successful repression of Tat induced macrophage migration has also been shown to involve CB2 receptor^20^. We wanted to investigate the differential effect of Tat variants on Müller glia and its ability to attach PBMCs. To investigate whether variation in Tat B and Tat C can affect PBMC attachment to Müller glia in the presence of AEA, we have performed experiments with antagonists against the CB receptors and inhibitors of MKP-1 or MEK-1. MIO-M1, the human Müller cell line, transfected with either Tat variants are further treated with the antagonists. Attachment of PBMCs is then investigated after 1 hour of incubation. Adhesion increases on treatment with Tat B/C transfected cells, in comparison to un-transfected cells (Fig. 8b). Inhibition by Ro-318220 blocking MKP-1, in Tat B and Tat C cells reduces adhesion, in comparison to Tat alone. The blocking of MEK-1 with U0126 shows dramatic change in Tat C transfected cells but not in Tat B cells. Using CB antagonists, AM-251 and AM-630 in Tat-transfected cells, lead to fall in adhesion. Engaging CB2 receptor with AM-630 in Tat transfected cells shows greater repression of PBMC attachment. AEA suppresses adhesion of PBMCs to Müller glia transfected with either Tat variant. Blockage of either CB1 or CB2, with AM-251 or AM-630, respectively in Tat + AEA cells, increases adhesion of PBMCs, compared to Tat + AEA but shows little difference between the two CB antagonists. Blocking MKP-1 in Tat B + AEA cells results in PBMCs adhering to MIO-M1, to significantly increase in comparison to Tat B + AEA cells. This emphasizes the role of MKP-1 in Tat B cells exposed to AEA. In contrast, MKP-1 inhibition in Tat C + AEA cells has negligible difference from control Tat C + AEA cells. MEK-1 inhibition by U0126 elevates adhesion of PBMCs for both Tat variants in comparison to Tat + AEA. Noticeably, this effect is more pronounced in Tat C + AEA cells. While all the antagonists are able to suppress PBMC attachment in Tat-exposed Müller glia, lack of adhesion is greater with AEA. These results suggest that engagement of either CB receptors may be useful in suppressing PBMC adhesion to Müller cells. Adhesion is mediated in Tat B + AEA cells overwhelmingly through MKP-1. In Tat C + AEA cells, MEK-1 is the primary regulator of PBMC attachment to Müller cells. The control experiments emphasise that the blockers and the empty plasmid cannot induce PBMC adhesion to MIO-M1 (Fig. 8a).

In summary, we find that HIV-1 Tat B and Tat C have distinct modes of action in inducing cytokines. AEA has the ability to suppress pro-inflammatory cytokines and induce higher expression of anti-inflammatory ones in Müller glia activated by either Tat protein. In Müller glia exposed to Tat B, AEA influences the switch in favour of anti-inflammatory cytokines through MKP-1, TAB2, pIκB and TTP. In marked contrast, Müller glia transfected with Tat C respond to AEA through MEK-1 and IRAK1BP1. The pivotal role of MKP-1 and MEK-1, and their respective importance in activation by Tat B and Tat C, is also reflected in PBMC attachment to Müller glia. This may have pharmacological importance for BRB protection during retinal inflammation.

## Discussion

The majority of HIV-1 infection may be attributed to clades B and C. Clade specific differences in Tat are responsible for neurodegeneration and glial activation^38^. In this study we show that the disparity in innate immune response by Tat B and Tat C can be explained by differential effect on signal transduction pathways involved in cytokine production. Furthermore, we report the novel observation that the ability of AEA to create a pro-survival milieu in Tat-activated Müller glia is differentially regulated in clades B and C. In concurrence with previous studies on neurons^3^, we find that Müller cell death is more on Tat B transfection than with Tat C. Triggering of cell death could be attributed to higher levels of activated caspase-3 and PARP in Tat B cells. AEA is able to partially reverse effects of either Tat. Activation by Tat which is marked by increase in GFAP also entails secretion of an array of cytokines. Tat B unequivocally up-regulated all the pro-inflammatory cytokines studied by us more than Tat C. It has been conclusively shown that greater cytokine production in Tat B, including anti-inflammatory IL-10 can be largely linked to the dicysteine motif, C30C31. Indeed, Tat C carrying this dicysteine motif is also more neurovirulent^8^. Addition of AEA selectively upregulates anti-inflammatory cytokines, IL-10 and TGF-β, and down regulates all the pro-inflammatory ones. Earlier work from the authors had pinpointed the MAPK pathway as a key component of innate immune response in Müller glia^15^. Both Tat variants lead to phosphorylation of MAPK components and their subsequent suppression on exposure to AEA. The crucial role of MAPK in inducing cytokines is confirmed by measuring them at the protein level after inhibitor studies. Cytokine production is regulated by a delicate balance maintained by feedback loops such as JAK-STAT1α and SOCS^29^. We report that Tat B and Tat C have very different effects on STAT1 and SOCS. Paradoxically, SOCS1 is more in Tat B than in Tat C glia. Concurrently, STAT1 levels are lower in Tat B cells compared to Tat C cells. At the later time points SOCS3 is higher in Tat C cells than in Tat B glia. It may be that other SOCS family members are similarly differentially affected. Previous reports^39^ indicate that SOCS3 expression induced by Tat B inhibits anti-viral interferon signalling to enhance HIV-1 replication in macrophages and microglia. It remains to be seen whether this differential action by the variant Tat proteins could be responsible for promoting progression toward neurodegeneration by allowing HIV-1 to evade protective host immune response. AEA action induces both STAT1 and SOCS to fall in glia activated with either Tat variants. In order to dissect the mechanistic basis of cytokine regulation we further investigate the upstream regulators of MAPK, MKP and MEK. Tat transfection with either variants increase expression of both MEK and MKP-1 with time. Application of AEA in activated glia reveals the differential response of the two Tat variants on cytokine production machinery. While in Tat B + AEA glia there is conspicuous increase in MKP levels, in Tat C + AEA it is MEK levels which show dramatic rise. Inhibition of MKP or MEK also reflects at the cytokine mRNA and protein levels, suggesting that the role is seminal only in activated Müller glia. Thus, in Tat C cells AEA acts to promote a beneficial milieu through MEK, but in Tat B activated glia MKP is the more important regulator of MAPK phosphorylation and subsequent control of cytokine generation. MKP-1 gene sequence is known to have enhanced association with Phospho-histone H3 in LPS-activated microglia on addition of AEA^40^. It is possible that the Tat variants may affect mkp-1 phospho-H3 interactions. The association between cytokines and signalling proteins extend at multiple levels of signal transduction, including transcription factors. As a master regulator of pro-inflammatory genes, the NF-κB signalosome is known to be activated in HIV-1 infection^41^. Enhanced transcriptional activity of the p65 subunit on exposure to HIV Tat has been reported. Tat B is known to enhance gene transcription by physically affecting both p65 as well as IκB^42^. We show that control of the NF-κB machinery is differentially regulated by Tat B and Tat C. Tat B increases phosphorylation of IκBα enormously, much more than in Tat C. This may be suggestive of Tat C interaction with p65 subunit of NF-κB but not IκBα. Addition of AEA cause pIκBα levels to fall. In our study, we observed partial abrogation of IkBα phosphorylation and consequent NF-κB activation on addition of MAPK inhibitors. ERK seems to be responsible for the low levels of pIκBα seen in Tat C cells. Simultaneously, we find that IRAK1BP1, an associated protein which acts as negative regulator of overwhelming cytokine response by modulating p65/p50 levels, is highly up-regulated in the presence of Tat C but not Tat B. AEA exposure in activated Müller glia increases IRAK1BP1 level, thus consolidating an anti-inflammatory environment. TAB2, an adaptor protein linked to Smad7^43^, which prevents NF-κB activation, increases significantly in the presence of both Tat B and Tat C. AEA down regulates TAB2 level in activated cells. TTP is known to aid in degradation of ARE containing mRNA transcripts of cytokines and affecting their stability. Manifold increase in TTP is observed in Tat C transfected glia, suggesting that inflammation could be restricted because of degradation of pro-inflammatory cytokine transcripts. AEA induces a rise in TTP in Tat B cells and thus may destabilise pro-inflammatory cytokine transcripts.

We conclude that while Tat B and Tat C both induce pro-inflammatory response, the milder neurotoxic effect of Tat C can be explained by the predominant role of MEK-1/2 rather than MKP-1. Differential effect on multiple proteins in the NF-κB transcription complex may also contribute to negatively regulating inflammation. A pro-inflammatory milieu can act as chemotactic signal to peripheral immune cells. The functional relevance of clade specific activation by Tat in Müller glia appears to be most salient in the course of disease progression. Existing reports suggest that a CB2 dependent mechanism may offer means to control HIV-1 infection in macrophages^44^. Reports have already linked Tat B activation to CB2 in macrophages^20^. CB2 dependent attenuation of leukocyte-endothelial cell interaction has previously emphasised the role of CB receptors in LPS-induced encephalitis^21^. The two Tat variants have different capacities to attach PBMCs. AEA can reduce leukocyte adhesion. In human Müller cells it appears that both CB receptors are involved in mediating PBMC attachment. This suggests that AEA may act as an immune-modulator through processes apart from interfering with chemokine production. The relative importance of MEK-1 in Tat C transfected cells and MKP-1 in Tat B cells are amplified in Tat + AEA cells, where specific antagonists can reverse AEA-mediated suppression in PBMC attachment. It remains to be seen how AEA can affect adhesion molecules which contribute to transmigration and HIV infectivity in different clades. Understanding the mechanism that induces inflammation dependent loss of CNS integrity will be essential in any attempt to manipulate neuro-inflammatory processes. It is likely that HIV-1 clades which possess different biological properties may cause differences in the progression of the disease pathogenesis in the eye. Such differences have been demonstrated in HIV-associated neurodegeneration between HIV clades B and C^45^,^46^. This study offers clues on biological functions of Tat in different clades in the context of the retina. While there is no evidence to indicate that HIV-1 infects Müller cells, it is possible that activated Müller cells aggravate retinal microglial infectivity by HIV-1 as it is known that microglia possess receptors to which HIV-1 can bind^44^.Our study further dissects the effect of the endocannabinoid AEA known to ameliorate inflammatory pathology. Evidence from this work emphasise that anandamide based intervention, while alleviating inflammation, is HIV-1 clade-dependent. The data presented here suggest that AEA can reduce neuro-inflammation induced by HIV-1 Tat. It thus holds promise as a potentially important adjunct therapeutic strategy for future consideration.

## Methods

Methods are in accordance with approved guidelines. All experimental protocols were approved by the ethics committee of Vision Research Foundation Institutional Review Board. The committee agreed and confirmed that the study was acceptable and under the general principles of research and in accordance with the Helsinki Declaration.

### Reagents and antibodies

Chemicals were purchased from the following sources: DMEM, Fetal bovine serum (FBS) (PAN-Biotech); DMEM F12, B27, N2 supplement, Glutamine (Invitrogen); Penicillin-Streptomycin, Trypsin (0.25%), EDTA (0.02%) solution (HiMedia); Endocannabinoids AEA (Sigma-Aldrich); Cannabinoid receptor antagonists/inverse agonists, AM-630 and AM-251 (Tocris); MAPK inhibitors; 3-[3-[2,5-Dihydro-4-(1-methyl-1H-indol-3-yl)-2,5-dioxo-1H-pyrrol-3-yl]-1H-indol-1-yl]propyl carbamimidothioic acid ester mesylate (Ro-318220), 1,4-Diamino-2,3-dicyano-1,4-bis[2-aminophenylthio]butadiene (U0126), 5-(2-Phenyl-pyrazolo[1,5-a]pyridin-3-yl)-1H-pyrazolo[3,4-c]pyridazin-3-ylamine (FR180204), Anthra[1-9-cd]pyrazol-6(2H)-one (SP600125), 4-[5-(4-Fluorophenyl)-2-[4(methylsulfonyl)phenyl]-1H-imidazol-4-yl]pyridine) (SB203580), 2-(4-Morpholinyl)-8-phenyl-4H-1-benzopyran-4-onehydrochloride) (Ly294002) (Cell Signalling Technology); Acrylamide, Ammonium persulfate, TEMED, SDS, Glycine, EDTA, TRIS and Tris-hydrochloride, Methanol (Sisco Research Lab). HIV1 Tat clade B expression vector (p229.1 B-Tat CC wild type) and HIV-1 Tat clade C expression vector (p214. C-Tat CS wild type), were gifted by Prof. U. Ranga, JNCASR, India and referred to as Tat B and Tat C throughout the manuscript. pEGFP and pcDNA3.1 vectors were purchased from Clontech and Invitrogen respectively. The following antibodies were obtained from Sigma; mouse monoclonal anti-β-actin (1:800), mouse polyclonal Anti-IRAK1BP1 (1:100). Rabbit polyclonal Bcl-2 (1:300), Cleaved Caspase-3 (1:500), total Caspase-3 (1:500), anti-phospho-ERK1/2 (1:100), total ERK1/2 (1:100), anti-phospho- SAPK/JNK (1:75), total SAPK/JNK (1:75), anti-phospho-p38 (1:100), total p38 (1:100), anti-phospho-Akt (1:50), total Akt (1:50), anti-phospho-JAK/STAT (1:100), total JAK/STAT (1:100), rabbit polyclonal SOCS-1 (1:100), Mouse monoclonal SOCS-3 (1:300), rabbit monoclonal anti-phospho-MEK (1:100), total MEK (1:100), Phospho-NF-κB p65 (1:100), NF-κB p65 (1:100), phospho-Iκ-Bα (1:100), total Iκ-Bα (1:75), TAB2 (1:500) antibodies were purchased from Cell Signalling Technology. Antibodies against mouse monoclonal MKP-1 (1:100), HIV-1 Tat (1:100), rabbit polyclonal MKP-2 (1:100), TTP (1:150), were obtained from Santa Cruz Biotechnology.

### Müller glia culture

For primary human Müller cell culture, cadaveric human eyes from normal donors of various ages (21 to 74 years) who had no history of ocular disease were collected from C.U Shah Eye Bank, Sankara Nethralaya, Chennai. The cadaveric eyes are collected after obtaining consent from all the donors or the next of kin for use of these samples in research by the C U Shah eye bank Sankara Nethralaya according to established guidelines; www.sankaranethralaya.org/eye-bank.html‎. All studies involving cadaveric human subject’s eyeballs were conducted in accordance with the guidelines of the World Medical Association's Declaration of Helsinki 2008. This project was reviewed and approved by the ethics committee of Vision Research Foundation Institutional Review Board. Müller glia was isolated according to standard protocol^47^ with minor modifications^15^. Cultures were 95% pure for Müller glia as tested by immunofluorescence.

MIO-M1 was gifted by Prof G.A. Limb. Cells were cultured in Dulbecco’s modified Eagle’s medium (DMEM) with 4.5 g/ L Glucose, 2 mM L- glutamine, 1 mM sodium pyruvate, 3.7 g/L NaHCO_3_, 100 IU/ml Penicillin, 100 μg/ml Streptomycin and 1 mM EDTA^48^.

### Cell Treatments

Human primary retinal Müller glia of 18–21 days old was used in all experiments. AEA was used at 10 μM. 4 μg of plasmid HIV-1 Tat B or Tat C was used to transfect Müller glia using Lipofectamine™ 2000 as recommended by the manufacturer in every experiment except in MTT, where 0.2 μg was added. AM-251 and AM-630 were used at 1 μM. Ro-318220, U0126, FR180204, SP600125, SB203580, and Ly294002 were used at 10 μM. HIV-1 Tat B or Tat C plasmid transfected cells (see Supplementary Fig. S1) are referred to as Tat B or Tat C cells throughout the manuscript. Tat released from Tat expressing cells such as astrocytes^49^,^50^, and neurons^10^ are known to mimic conditions and levels of the protein seen in the vicinity of HIV-infected cells in patients. For MTT assay, Müller glia were plated in 96-well culture plates at density of 8 x 10^3^cells/well and allowed to adhere at 37 °C overnight. In all experiments with transfected cells, Tat B or Tat C plasmids were incubated for 24 hours before further treatments. Control cells comprised of (1) untreated, untransfected primary cultures (2) cells transfected with the empty plasmid (pcDNA 3.1) and undergoing no treatment. In some experiments Tat B and Tat C transfected cells without treatment were used as additional controls. Cells were incubated with either AEA alone or coincubated with AM-251or AM-630 for 24 hours to perform MTT assay. For isolation of total cell RNA and subsequent reverse transcription PCR or Real-time PCR (qPCR), Müller glia were plated in 6-well plates at density of 1.8 x 10^6^ cells/well and allowed to adhere at 37 °C overnight. Plated Müller cells were transfected with either Tat B or Tat C plasmid alone. After transfection cells were incubated with AEA or AM-251, AM-630, for 8 hrs for the majority of cytokines or 10 hours for CXCL-10 and MCP-1. For ELISA, one hour before each experiment, media containing serum were removed and replaced with serum-free media. After Tat B or Tat C plasmid transfection, cells were incubated with AEA, or coincubated with AM-251, AM-630, together with any one of the following blockers; FR180204, SP600125, SB203580, and Ly294002 for 24 hours to measure cytokine levels. Western blotting was used to study phosphorylation and changes in activation. Transfected cells were treated with AEA alone or coincubated with AM-251, AM-630 as given below. Treated cells were lysed at 0, 30 minutes, 1, 2, 8 and 24 hours to probe for phosphorylated as well as total ERK1/2, JNK, p-38, AKT, STAT1-α, SOCS-1, and SOCS-3. To probe for MEK-1 and MKP-1 Tat transfected cells were coincubated with AEA for 0, 2, 6, 8, and 24 hours and subsequently lysed. In inhibitor experiments with MKP-1 and MEK-1 blockers, transfected cells were incubated with AEA alone or coincubated with Ro-318220 or U0126 for 0, 2, 8, and 24 hours and lysed for probing against phosphorylated and normal ERK1/2, JNK, p38. For the expression analysis of NF-κB, p-IκBα, TAB-2, TTP and IRAK1BP1, transfected cells were treated with AEA or coincubated with AM-251, AM-630 for 24 hours and lysed for probing with the respective antibodies.

### MTT Viability Assay

MTT assay according to manufacturer’s instructions was used to measure cell viability. Percentage cell viability was obtained after normalization (Test OD/Control OD X 100).

### Knockdown of MKP-1 and MEK-1 by siRNA

siRNA transfections were performed using RNAi-MAX Lipofectamine™ 2000 (Invitrogen, Carlsbad, CA) 48 hours prior to Tat B or Tat C plasmid transfection. 4 x 10^5^ cells plated on six-well dishes were transfected with MKP-1 siRNA (sc-35937), control scrambled MKP-1 siRNA (sc-37007), MEK-1 siRNA (sc-29396) and control scrambled MEK-1 siRNA (sc-44230) (Santa Cruz Biotechnology Inc.). 48 hours after transfection, cells were treated with Tat B, Tat C or Tat B + AEA, Tat C + AEA for 24 hours. Cells were lysed and used for Western blot analysis for MKP-1/MEK-1 knockdown, total RNA extraction for cytokine levels, and the same culture medium was collected for cytokine measurement by ELISA.

### qPCR quantitation of mRNA

2 μg of RNA was extracted from primary Müller glia using the Qiagen RNeasy Kit according to the manufacturer’s instructions (Invitrogen). Cells were either untreated or treated with Tat B or Tat C alone and/or Tat B + AEA/Tat C + AEA for 8 or 10 hours. Quantitative analysis of TNF-α, IFN-γ, TGF-β, IL-6, IL-10, IL-12p40, CXCL-10, and MCP-1 mRNA was done by qPCR using the SYBR Green detection method. Primers and reaction conditions were from the following references: for IL-10, IFN-γ, and TGF-β, IL-12p40^51^, MCP-1^52^, CXCL-10^53^, IL-6, 18S ribosomal RNA^54^,^55^ respectively. After thermal cycle, the melting point was assessed and dissociation curves were generated to confirm the specificity of the reaction. Data were normalized using Ct values for the internal control gene 18S RNA in each sample. The gene expression in the treated sample was calculated relative to the untreated control^56^,^57^. The fold change in gene expression (differences in changes in Ct value) was then determined as log2 relative units.

### Western blot analysis

Extractions of nuclear and cytoplasmic proteins were according to^58^. 50 μg protein was loaded on SDS-PAGE gels and transferred to a nitrocellulose membrane. Membranes were blocked, and then probed with appropriate antibodies. Tetraethyl benzidine/Hydrogen peroxide (TMB/H_2_O_2_) reagent^59^ was used according to the manufacturer’s instructions for detection. The blots were scanned and densitometric analysis was performed with ImageJ (NIH), and the intensity for each band was normalized to the intensity of the corresponding total ERK1/2, JNK, p-38, AKT, SOCS1, JAK/STAT, MEK, MKP, NFκ-B, IκBα or β-actin band. For every protein probed by Western blot, gels have been run under the same experimental conditions. Background normalization was also performed.

### Measurement of cytokines by ELISA

Primary Müller glia was treated as described in Cell treatments and their supernatant collected. IL-10 cytokine levels in supernatants were quantified using specific ELISA kit purchased from R&D Systems (Minneapolis, USA), according to manufacturer’s instructions. TGF-β, TNF-α, IFN-γ, IL-6, IL-12p70 was quantified using specific ELISA kits (Quantikine Human Immunoassay) according to manufacturer’s instructions. The assay sensitivity and the intra- and inter-assay coefficient of variations were according to manufacturer’s instructions.

### Adhesion Assay

For the adhesion assay, MIO-M1 cells were plated in 96 well plates. MIO-M1 cells were transfected with Tat B or Tat C for 24 hours before further treatment. All MIO-M1 treatments were for 24 hrs. PBMC isolation was according to established protocol^60^. Freshly isolated PBMCs were loaded with 5 μM of Calcein-AM, a fluorescent dye for living cells. After 30 minutes of Calcein-AM incubation, PBMC were washed for unincorporated dye, and then adjusted to 5 × 10^4^ cells/ml per well. MIO-M1 supernatant was removed, washed twice with PBS prior to the addition of 5 × 10^f^ PBMC/500 μl, pre treated with Calcein-AM to each cell. Cells were incubated for 1 hr and then washed very carefully four times with pre-warmed PBS. Attachment of PBMC was ascertained under a fluorescent microscope. Fluorescence was determined using SpectraMax at 485/515 nm. Fluorescence from adhering cells to MIO-M1 without Tat plasmids or any other treatment was used to normalize. Amount of fluorescence from PBMC binding to the MIO-M1 were represented as arbitrary values.

### Statistical analysis

Results are expressed as mean ± S.E. of at least three experiments performed with cell preparations from different dates. Statistical significance among groups were determined by one-way analysis of variance followed by Bonferroni post hoc analysis. Statistical analysis of the data was performed using SPSS version 12.0 software (Chicago, IL). Probability value p < 0.05 was considered significant.

## Author Contributions

N.C conceived and designed the experiments. G.K performed the experiments. Both N.C and G.K contributed to reagents, materials and analysis tools. N.C and G.K both contributed to manuscript and figure preparation.

## Additional Information

**How to cite this article**: Krishnan, G. and Chatterjee, N. Differential immune mechanism to HIV-1 Tat variants and its regulation by AEA. *Sci. Rep.* **5**, 09887; doi: 10.1038/srep09887 (2015).

## Supplementary Material

Supplementary Information
