# Supplementary material for: 1Differential immune mechanism to HIV-1 Tat variants and its regulation by AEA
Source: Sci Rep. 2015 May 6;5:9887. doi: 10.1038/srep09887 (PMC4421801; doi:10.1038/srep09887)
Supplement: Supplementary Information [file srep09887-s1.pdf]

Title: Differential immune mechanism to HIV-1 Tat variants and its regulation by AEA

Authors: Gopinath Krishnan M.Sc.[1, 2], Nivedita Chatterjee PhD[1*]

[1*]L&T Department of Ocular Pathology, Vision Research Foundation, Sankara Nethralaya, 41 College Road, Chennai, India 600006. [2] Research Scholar, CeNTAB, School of Chemical and Biotechnology, SASTRA University, Tanjore, India.

Running Title: HIV-1 clade specific effects on Müller glia

To whom correspondence should be addressed: *Nivedita Chatterjee, L& T Department of Ocular Pathology, Vision Research Foundation, Sankara Nethralaya, 41 College Road, Chennai, India 600006. Telephone - 0091 44 28233556. Fax- 0091 44 28254180, Email:chatterjee.nivedita@gmail.com

Supplementary Figure Legends

Supplementary Figure 1. AEA show dose- and time-dependent regulation of production of TNF-α in Müller cells on transfection of Tat plasmids.

Representative cropped Western blot image on probing with HIV-1 Tat antibody shows the expression of Tat B and Tat C protein on transfection of Tat B or Tat C plasmids. Full length blots are shown in Supplementary Figure S6. Cells transfected with empty plasmid or control cells without transfection did not show expression of HIV-1 Tat of either variant (A). Representative microscope images of primary Müller cells co-transfected with GFP and Tat C (B). 4 ug of GFP plasmid was co-transfected in Müller cells with 4 ug of either Tat plasmid. Fluorescence for GFP as measured with FACS showed on an average 55% transfection efficiency (C). ELISA measurements show TNF-α level fall with increasing concentrations of AEA (1, 5, 10, and 20 μM) in cells transfected with Tat B/C plasmid (D, E). Time kinetics of TNF-α in Tat B and Tat C cells at mRNA (F) and protein (G) levels show highest values at 8 hours and 24 hours, respectively. Cells transfected with empty plasmid or 10 μM AEA alone do not induce TNF-α cytokine production (H). Time kinetics of TNF-α on empty plasmid transfection or AEA alone show no TNF-α production at all time points studied (I). Results shown are the mean ± S.E (n=3). $*p<0.05$, $**p<0.01$, $***p<0.001$ vs. Control.

Supplementary Figure 2. Effect on signal transduction components of empty plasmid transfection or treatment with AEA.

Western blot image shows that empty plasmid transfection or AEA alone do not affect ERK1/2 phosphorylation, at all time points studied (A). Phosphorylation of STAT-1α, SOCS-1, and

SOCS-3 expression do not change upto 24 hours on transfection of empty plasmid or AEA alone (B). Phosphorylation of MEK1/2, MKP-1 and MKP-2 are not induced at all time points studied on transfection with empty plasmid or AEA alone (C). Nuclear NF-κB, pIκB, IRAK1BP1, TAB2, and TTP expression do not increase on transfection with empty plasmid or AEA, and the CB blockers alone. pIκB expression does not increase on treatment with MAPK blockers (D). Representative cropped immunoblots are shown. Full-length blots are presented in Supplementary Figure S6. Results shown are the mean ± S.E (n=3).

Supplementary Figure 3. pAKT was regulated by both Tat B/C

Tat B and Tat C cells showed significant phosphorylation of PI3K/AKT signalling pathway. Addition of AEA significantly dephosphorylated pAKT for both Tat B/C. The addition of either AM-251 or AM-630 abrogated effects of AEA on the Tat B cells partially and almost completely in Tat C cells. ***p<0.001 vs. Control. $$$p<0.001 vs Tat B. &&&p<0.001 vs Tat C. #p < 0.05, ###p < 0.001 vs. Tat B+AEA. +p<0.5, vs. Tat C+AEA.

Supplementary Figure 4. AEA decreases pro-inflammatory cytokine production through MAPK, while increasing anti-inflammatory cytokines, in Müller glia activated by both Tat variants.

AEA induced production of IL-10 (A) was reversed by inhibition of ERK1/2, JNK, p38 with FR180204, SP600125, SB203580, respectively. TNF-α (B), IL-6 (C), IL-12p70 (D), CXCL-10

(E) and IL-8 (F) show reversal of AEA induced suppression on blocking the same pathways. Note that values are higher than those of either Tat+AEA only. PI3/AKT mediation in production of IL-6 (C), CXCL-10 (E) and IL-8 (F) in the presence of AEA is more pronounced.

Results shown are the mean ± S.E (n=3). ***p<0.001 vs. Control. $p<0.001 $$p<0.01, $$$p<0.001 vs Tat B. &p<0.05, &&p<0.01, &&&p<0.001 vs Tat C. #p < 0.05, ##p<0.01, ###p < 0.001 vs. Tat B+AEA. +p<0.5, ++p<0.01, +++<0.001 vs. Tat C+AEA.

Supplementary Figure 5. AEA acts preferentially through MEK-1 in controlling cytokine mRNA production in Tat C activated Müller glia.

Anti-inflammatory IL-10 and inflammatory MCP-1 mRNA levels were measured at 8 and 24 hours on knockdown with either MKP-1 or MEK-1 siRNA. In Tat B+AEA cells, both cytokine levels change on MKP-1 knockdown (A-B). Tat B+AEA control cells induce similar levels of cytokines as in MEK-1 siRNA treated cells. In contrast, it is MEK-1 silencing which regulates IL-10 and MCP-1 in Tat C+AEA cells (C-D). Tat C+AEA control cells induce similar levels of cytokines as in MKP-1 siRNA treated cells. Results shown are mean ± S.E. (n=3).

Supplementary Figure 6. **Western blot images**

Full-length pictures of the blots presented as cropped panels in main-text figures

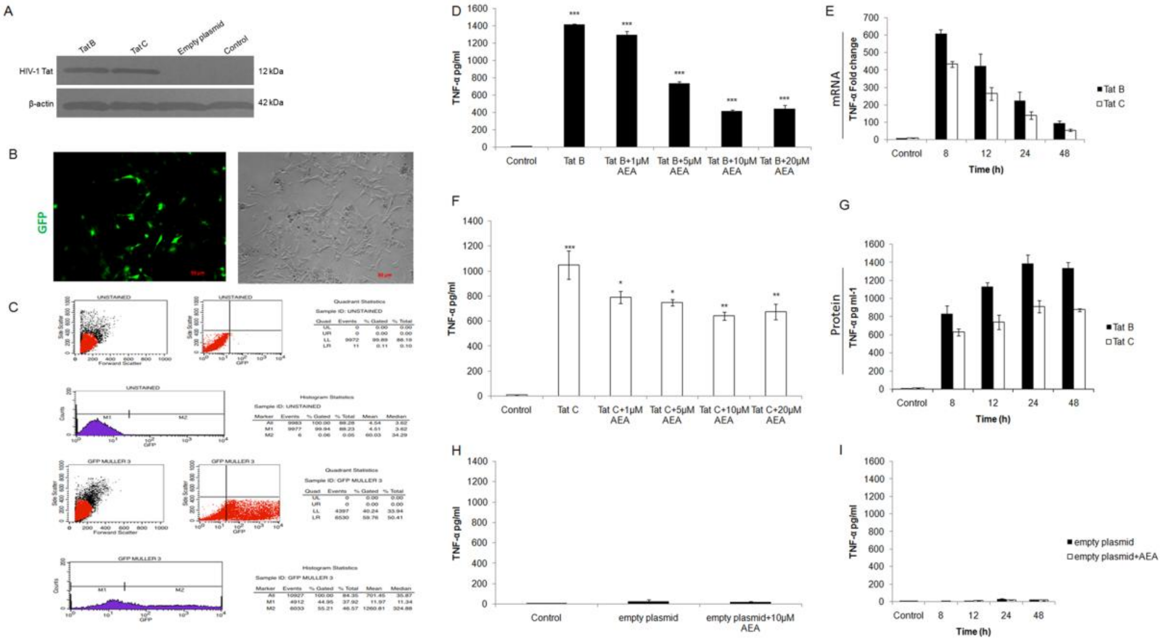
A
Tat B
Tat C
Empty plasmid
Control
HIV-1 Tat
12 kDa
β-actin
42 kDa
B
GFP
C
D
TNF-α pg/ml
Control
Tat B
Tat B+1µM AEA
Tat B+5µM AEA
Tat B+10µM AEA
Tat B+20µM AEA
E
mRNA
TNF-α Fold change
Tat B
Tat C
Time (h)
F
Tat C
Tat C+1µM AEA
Tat C+5µM AEA
Tat C+10µM AEA
Tat C+20µM AEA
G
Protein
TNF-α pg ml-1
H
empty plasmid
empty plasmid+10µM AEA
I
empty plasmid+AEA

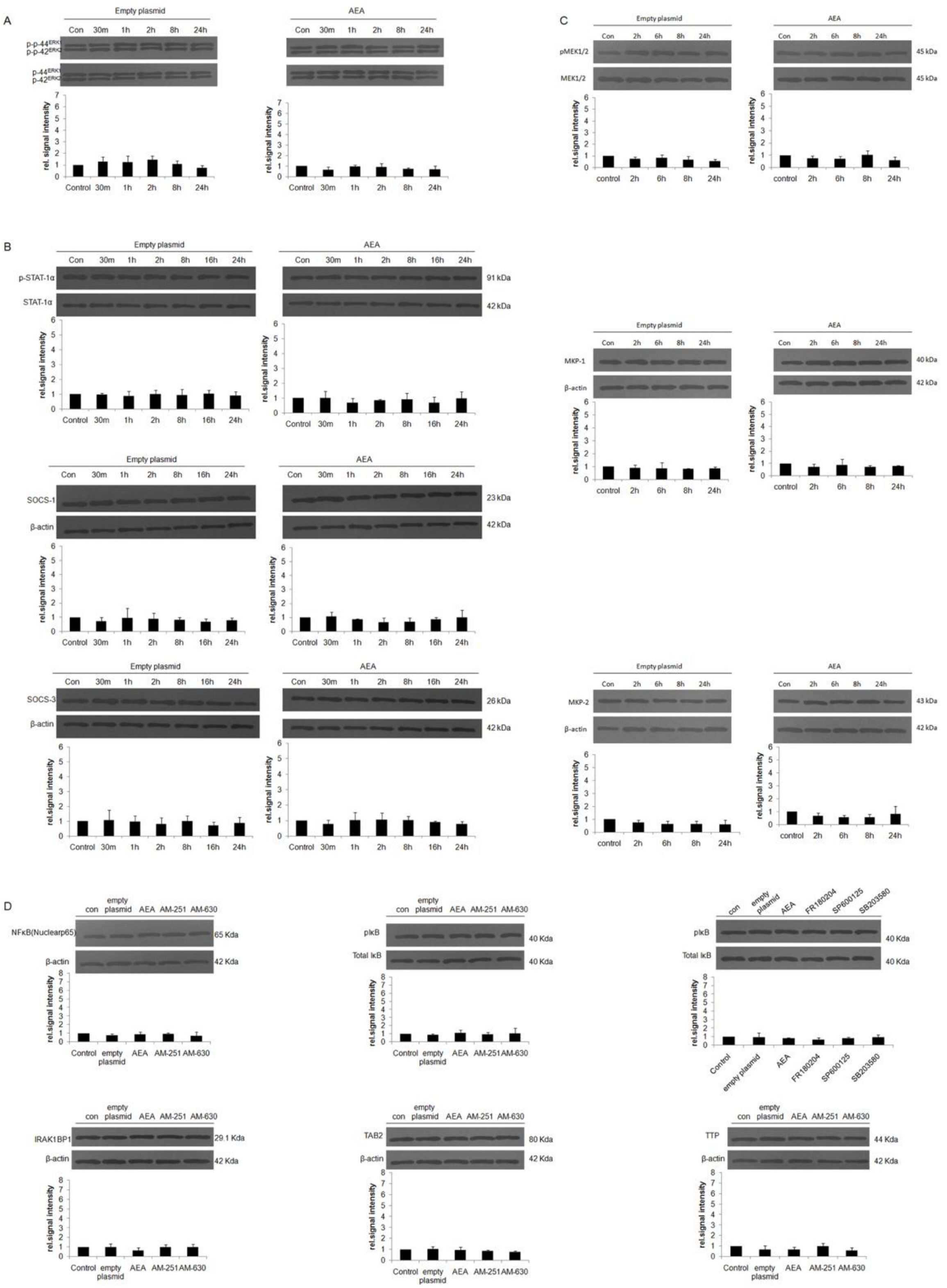
A
Empty plasmid
AEA
Con 30m 1h 2h 8h 24h
p-p-44ERK1
p-p-42ERK2
p-44ERK1
p-42ERK2
rel. signal intensity
Control 30m 1h 2h 8h 24h
C
Empty plasmid
AEA
Con 2h 6h 8h 24h
pMEK1/2
MEK1/2
45 kDa
control 2h 6h 8h 24h
B
Con 30m 1h 2h 8h 16h 24h
p-STAT-1α
STAT-1α
91 kDa
42 kDa
Control 30m 1h 2h 8h 16h 24h
MKP-1
β-actin
40 kDa
42 kDa
SOCS-1
23 kDa
SOCS-3
26 kDa
MKP-2
43 kDa
D
con empty plasmid AEA AM-251 AM-630
NFκB(Nuclearp65)
65 Kda
42 Kda
pIκB
Total IκB
40 Kda
con empty plasmid AEA FR180204 SP600125 SB203580
Control empty plasmid AEA AM-251 AM-630
IRAK1BP1
29.1 Kda
TAB2
80 Kda
TTP
44 Kda

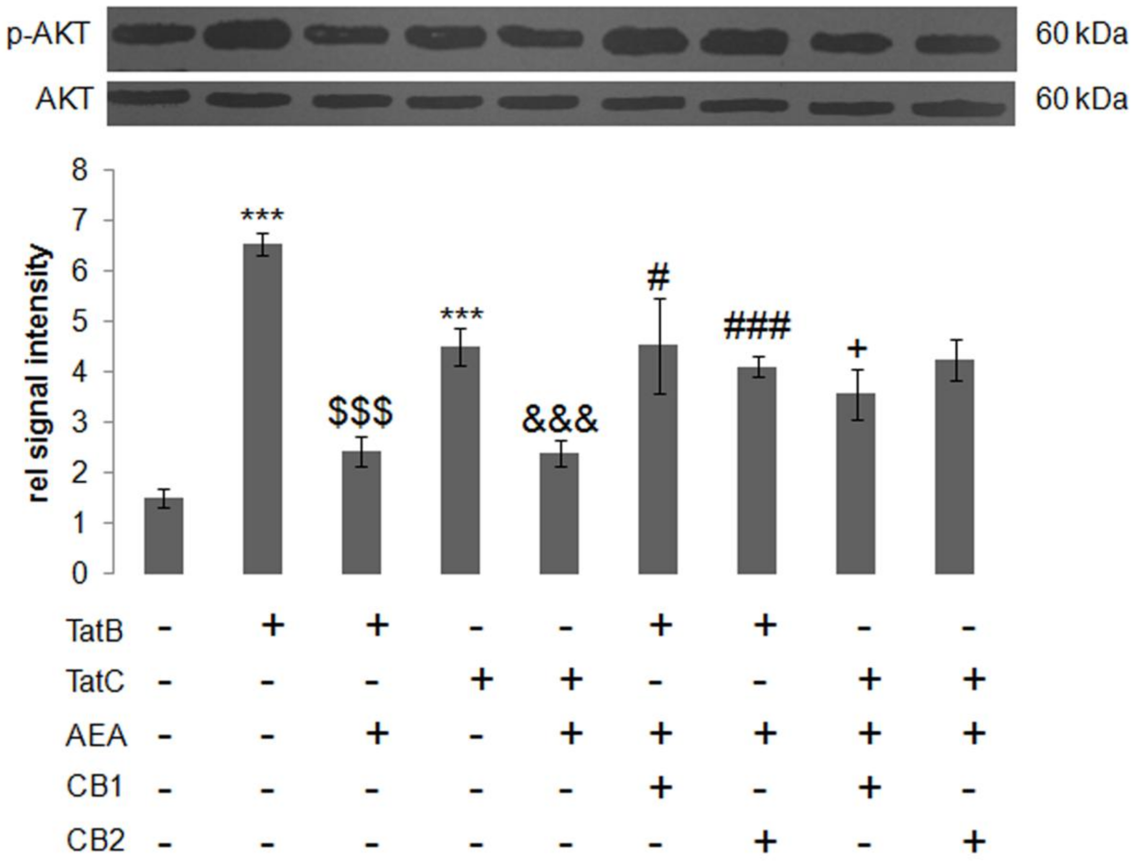
p-AKT
60 kDa
AKT
60 kDa
rel signal intensity
TatB
TatC
AEA
CB1
CB2

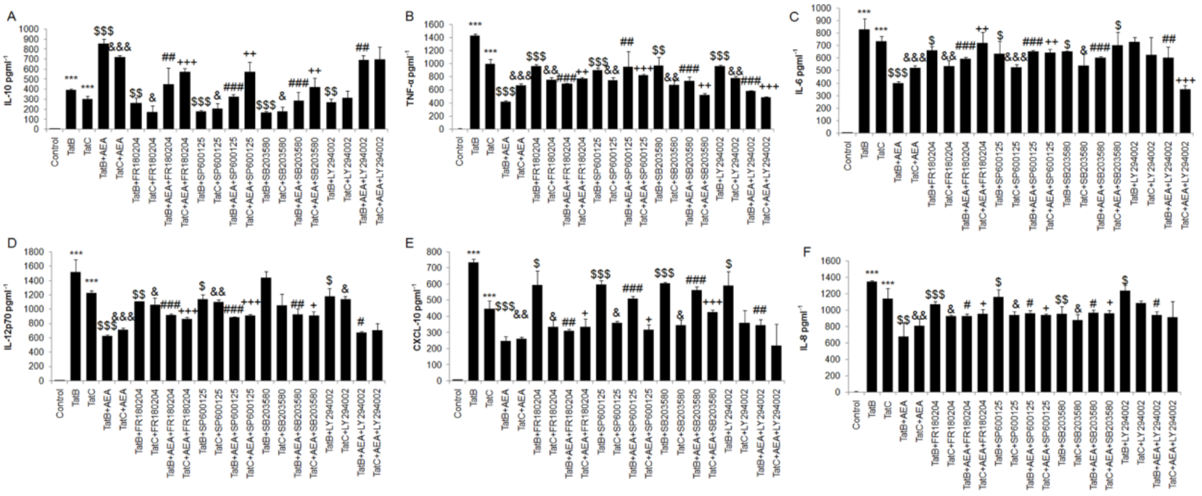
A
IL-10 pgml⁻¹
B
TNF-α pgml⁻¹
C
IL-6 pgml⁻¹
D
IL-12p70 pgml⁻¹
E
CXCL-10 pgml⁻¹
F
IL-8 pgml⁻¹

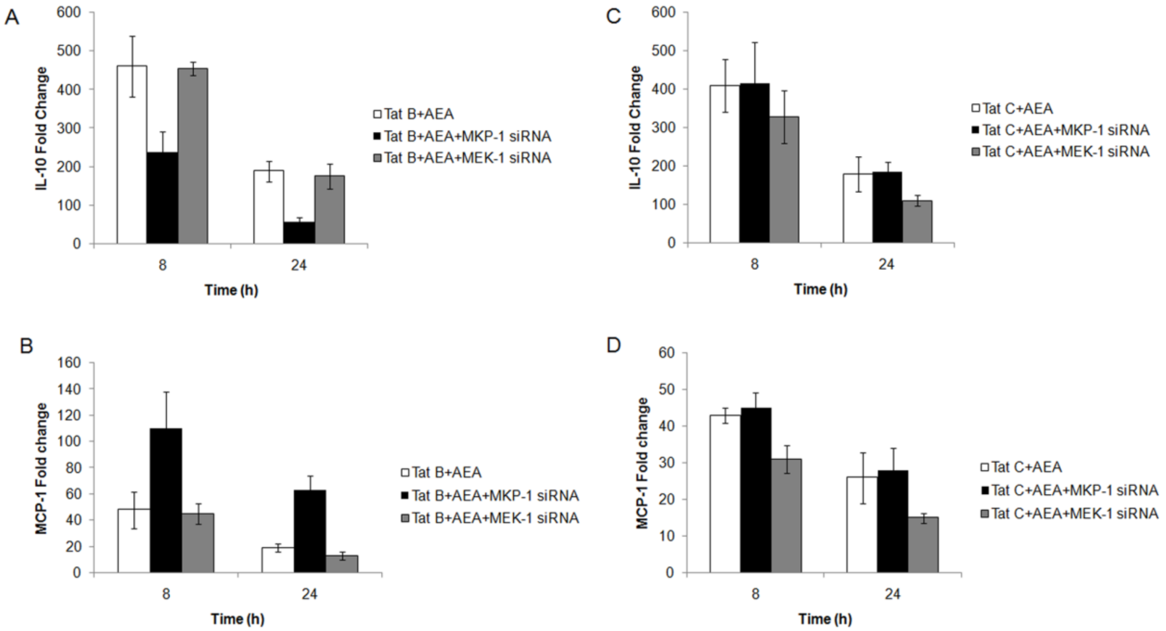
A
IL-10 Fold Change
600
500
400
300
200
100
0
8
24
Time (h)
Tat B+AEA
Tat B+AEA+MKP-1 siRNA
Tat B+AEA+MEK-1 siRNA
B
MCP-1 Fold change
160
140
120
100
80
60
40
20
0
8
24
Time (h)
Tat B+AEA
Tat B+AEA+MKP-1 siRNA
Tat B+AEA+MEK-1 siRNA
C
IL-10 Fold Change
600
500
400
300
200
100
0
8
24
Time (h)
Tat C+AEA
Tat C+AEA+MKP-1 siRNA
Tat C+AEA+MEK-1 siRNA
D
MCP-1 Fold change
60
50
40
30
20
10
0
8
24
Time (h)
Tat C+AEA
Tat C+AEA+MKP-1 siRNA
Tat C+AEA+MEK-1 siRNA

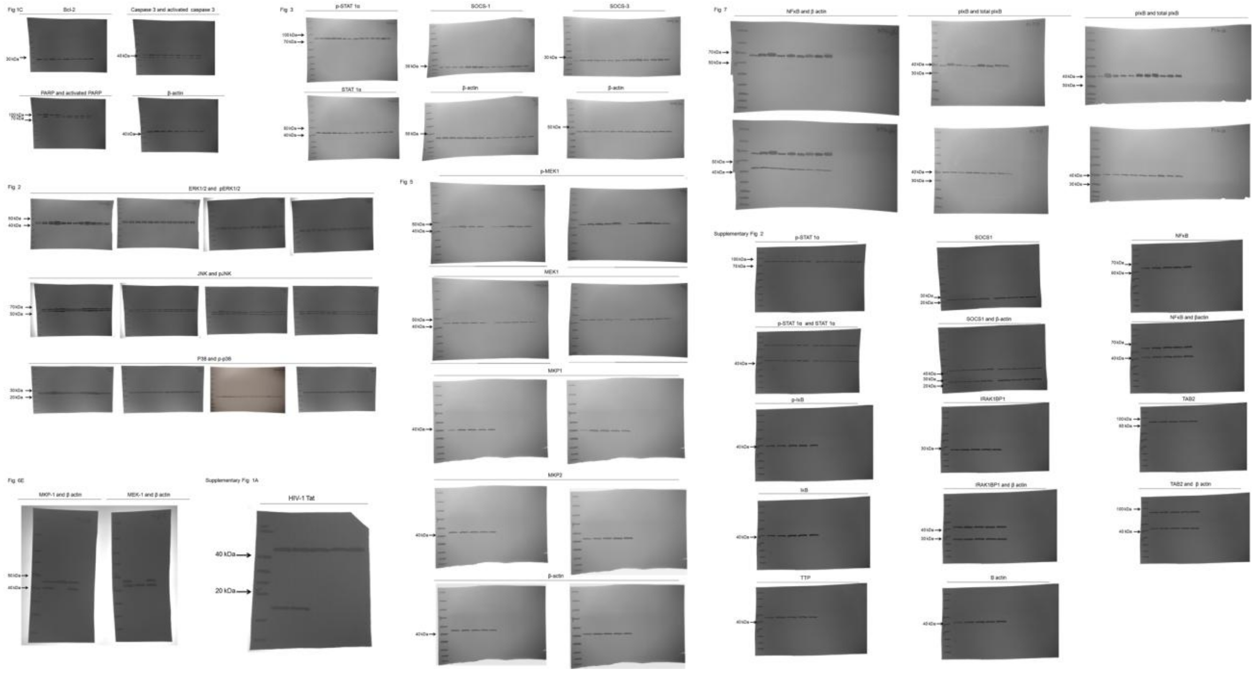
HIV-1 Tat
40 kDa
20 kDa
